# Productive and Reproductive Record Keeping in Low-to-Middle-Income Tropical Livestock Systems: Challenges and Perspectives

**DOI:** 10.3390/ani16111691

**Published:** 2026-05-31

**Authors:** Juan José Romero-Zúñiga, Carlos S. Galina, Mariana Geffroy, Manuel D. Corro, Martín Maquivar

**Affiliations:** 1 Programa de Investigación en Medicina Poblacional, Escuela de Medicina Veterinaria, Universidad Nacional de Costa Rica, Campus Benjamín Núñez, Heredia 40101, Costa Rica; juan.romero.zuniga@una.ac.cr; 2 Departamento de Reproducción, Facultad de Medicina, Veterinaria y Zootecnia, Universidad Nacional Autónoma de México, Ciudad Universitaria, México City 04510, Mexico; cgalina@unam.mx (C.S.G.); mgeffroy@comunidad.unam.mx (M.G.); 3 International Joint Laboratory Institut de Recherche pour le Développement/UNAM ELDORADO, Mérida 97069, Mexico; 4 Centro de Enseñanza Investigación y Extensión en Ganadería Tropical, Facultad de Medicina Veterinaria y Zootecnia, Universidad Nacional Autónoma de México, Tlapacoyan 93650, Mexico; macorro@unam.mx; 5 Department of Animal Sciences, Washington State University, Pullman, WA 99164, USA

**Keywords:** production efficiency, decision making, animal production, reproduction, sustainability, record keeping, global change

## Abstract

Tropical regions can vary according to geographical location, climatic conditions, socio-economic characteristics and the diversity of biological resources. In animal production systems in tropical regions, dairy cattle and/or dual-purpose farmers often struggle to maintain accurate records due to a multitude of factors. Low-to-middle-income farmers face challenges including financial constraints, limited access to veterinary services/technical consultation, limited awareness of available technologies, and the inability to implement them. These barriers hinder the adoption of effective herd management practices and preventive health measures to optimize production and health. Without proper record keeping, the limited information available may not accurately reflect the farm situation, and decision making could lead to adverse economic and environmental outcomes. Despite technological advancements that simplify data collection, analysis and interpretation, many tropical farms still encounter significant challenges in adopting these practices. The objective of the present scoping review is to provide an overview of the current status and to highlight the importance and necessity of collecting, maintaining, and interpreting records to achieve sustainable animal production systems in tropical regions.

## Simple Summary

There is a lack of reliable data from records in cattle farms in the tropics. This has limited adequate decision making by government bodies, professionals and farmers. There is, therefore, a pressing need for a simple and inexpensive system for registering on-farm data based on accurate information. Professionals working in the farm industry will benefit from accurate information, which, in turn, will assist them in their decision making. The objective is to shed some light on the shortcomings related to data recording. Nowadays, there is a demanding need to accurately record events; the economics of the small-to-middle-income farmers depend on improving their marginal gains, which can be the case in quite a few of their enterprises. The system of recording does not need to be complicated; it is a matter of convincing farmers of the importance of keeping up-to-date records in their establishments.

## 1. Methodology

This narrative review was developed from a structured search of the scientific literature on Web of Science and PubMed. The search aimed to identify publications addressing the use, limitations, and value of record keeping in cattle production systems under tropical conditions, with particular attention paid to low- and middle-income contexts. Tropical regions of the world, according to the ONU, are located in countries around the Equator, with temperatures ranging from 20 °C and above and high humidity [1]. For the context of this review and according to the world bank [2], we define low-to-middle-income farmers as those with a net annual income equal or lower to $4515 USD. Farmers in this region can be subdivided into those with medium-size farms, between 100 and 300 cows, and small-size farms, with less than 100 cows. The initial strategy combined broad terms related to records (defined as any quantitative or qualitative information collected on the animal enterprise), production environment, and system; we specifically select the references related to bovine species *Bos taurus* or *Bos indicus*, using the following search string: “record keeping” AND tropic AND (“dual-purpose” OR beef) AND cattle.

This search retrieved a limited number of publications, approximately 20 in Web of Science and 57 in PubMed. After screening the titles and abstracts, many records were found to be only marginally related to the review’s purpose. Some did not address record keeping as a tool for productive, reproductive, health, or management decision making; others focused on non-tropical systems or used the term “records” in unrelated contexts.

For this reason, the search strategy was expanded. Instead of relying solely on the general term “record keeping”, additional searches were structured around the husbandry and management domains, in which farm records are essential for monitoring, interpretation, and decision making. These domains included reproduction, milk and meat production, genetics, nutrition, animal health, farm economics, and sustainability. Search terms were combined using expressions such as “productive records”, “reproductive records”, “herd records”, “farm records”, “*Bos indicus*”, “*Bos taurus*”, “tropical cattle”, “dual-purpose cattle”, “dairy cattle”, “production efficiency”, “reproductive performance”, “herd management”, “decision-making”, and “sustainability”. This broader strategy helped identify the publications in which records were not necessarily the focus but were central to interpreting the outcomes related to productivity, reproduction, economics, or health.

To reduce the risk of excluding the relevant regional literature, the search was extended beyond Web of Science and PubMed to include complementary sources, particularly Latindex and SciELO for Latin American, Caribbean, Iberian, and South African scientific publications. Europe PMC (PubMed Central) and African Journals Online were also consulted when applicable. These sources were considered relevant because research on tropical livestock systems, farm management, and record-keeping practices may be published in regional journals but not fully represented in the main international databases. This complementary search was especially useful for identifying the literature from countries where dual-purpose cattle production, *Bos indicus* and *Bos taurus* crossbreeding, smallholder dairy systems, and tropical herd management are common.

The database and index searches were complemented with a snowballing strategy. The reference lists of selected publications were screened to identify additional sources not retrieved through the original search strings. When relevant, the articles cited within those publications were also examined to determine whether they contributed conceptual, empirical, methodological, or technical information related to the collection, maintenance, analysis, or interpretation of records within cattle production systems. This process continued until no substantially new thematic contributions were identified.

Sources were included when they addressed record use directly or when records were necessary for evaluating productive performance, reproductive efficiency, herd health, farm economics, sustainability, or evidence-based decision making under tropical or subtropical conditions. Publications focused exclusively on highly intensive temperate systems were considered only when their concepts, indicators, methods, or technological approaches were applicable to tropical cattle production.

The selected literature was analyzed qualitatively and organized thematically according to its contribution to understanding the role of records in evidence-based decision making, productive and reproductive performance, herd health, economic management, sustainability, and adaptation to global change.

## 2. Livestock and Global Change

The world’s population is estimated at 8.3 billion, and projections indicate that it will surpass 10 billion by 2050 [2]. This implies significant challenges in meeting demand for food, goods, and services from livestock production systems while minimizing environmental impacts and achieving sustainability.

Historically, countries with strong economies and advanced technology implemented at the farm enterprises have been concentrated primarily in countries located in temperate climates above the tropic of Cancer and below the tropic of Capricorn [3]. In contrast, many tropical countries face significant challenges, including systemic poverty, malnutrition of the population, and increased vulnerability to the impacts of global climate change. According to the United Nations [1], tropical regions host 43% of the world’s population and more than 80% of terrestrial biodiversity. Approximately 75% of this population lives in low-to-middle-income countries, which collectively account for 40% of global economic activity and nearly 60% of global CO_2_ emissions [3]. Additionally, the lack of infrastructure, socio-economic characteristics, and reduced capacity to adapt to environmental change makes tropical countries and their production systems vulnerable and makes it more difficult to meet animal protein demand, thereby hindering their efforts to become efficient, economically viable, and sustainable. In addition to these challenges, the implementation of effective husbandry practices such as record keeping, specialized nutritional diets, management and prevention of the health and reproductive technologies inevitably possess fluctuations in the efficiency of the farm.

Livestock production in the tropics is an important source of employment, income, food security, and a major source of high-quality protein essential for human development [4]. Despite hosting 80% of the world’s cattle, tropical regions have inefficient production units. Factors such as low technology transfer and adoption, management of the biological resources (such as land and water), low economic input, high environmental variability, low professional input from veterinarians, extensionists, or agronomists, and increasing environmental footprints [5,6]. Over the past 20 years, all livestock systems have contributed to greenhouse gas emissions, deforestation, water scarcity, and biodiversity loss [7,8,9,10]. In a recent paper, Manzano et al. [11] compared the greenhouse gas (GHG) emissions of pastoral livestock systems and wildlife-dominated pastures in Tanzania. Results from this study suggest that both wildlife and pastoralism have similar GHG emissions. Livestock grazing systems should promote soil and plant health, avoid overgrazing, and mimic the natural relationships among animals, plants, and the environment.

Producers with low-to-middle-incomes in tropical regions face dynamic economic conditions, high supply price volatility, and low returns on their products. In a review, Hsiang and Meng [12] suggest that climatic variability in tropical regions drives economic variation in these regions. Moreover, variations in temperature, humidity, and rainfall patterns directly affect agricultural activities, including crop yields, pasture growth, and, consequently, livestock production. As a result, high variability may help explain the low economic and productive growth observed in tropical countries.

Another critical aspect of livestock industries in the tropics is the limited technological development of these processes. Traditionally, producers in these regions face underdevelopment, low technology transfer and adoption, and the use of rudimentary and outdated agricultural practices that negatively impact the environment and regional biodiversity [13]. Furthermore, tropical livestock systems have been characterized by a lack of land management, causing the degradation of the soil and pastures as well as low productive efficiency of the vegetation, leading to undesirable socio-economic and environmental effects [14].

Ultimately, production systems must become socially, economically, and environmentally sustainable enterprises rooted in ethical practices to support global human and animal welfare and environmental demands [15]. Therefore, strategies must be implemented at the farm level to identify suitable management practices and maintain useful records to achieve sustainable animal production systems. One of the first steps towards these goals must be the implementation of clear, specific, action-oriented collection and interpretation of data as to drive tangible results for the farm. Due to the nature and high variability of tropical systems, these steps must be tailored to the needs and realities of each enterprise. Associations of producers and professionals willing to participate actively with clear communication channels and defined roles, fostering trust and direct collaboration, are imperative for success.

### 2.1. Characteristics of Cattle Systems in Tropical Developing Countries

Tropical cattle enterprises vary considerably in terms of natural, human, and economic resources available in these regions [16]. Nonetheless, the general management procedures for dairy and/or dual-purpose cattle rely heavily on extensive or semi-extensive conditions, characterized by low levels of technological adoption and seasonal management practices based on forage availability and rainfall patterns [17,18]. Cattle farms in these areas often raise Zebu breeds (*Bos indicus*) or *Bos taurus* × *Bos indicus* crosses, primarily for milk production. Alternatively, some farmers keep these animals as dual-purpose systems that produce mainly milk or dairy products and meat from male calves [19,20]. Farms in these regions are generally small and less technologically advanced, often relying on outdated management practices and minimal or non-existent records-keeping systems [21]. Furthermore, most farmers do not have a designed diet for their livestock to sustain production, as animal feeding relies on seasonal forage growth and grazing cycles of the region, which results in significant variability in reproductive and productive performance due to variations in forage quality and yield. In some instances, farmers supplement with concentrates only in specific phases of the production cycle, such as the pre-breeding and breeding season, early stages of development and growth and during the lactation of the females [20,22]. Farmers in these regions generally maintain between 1 and 50 animals [17,18]. Table 1 summarizes the key characteristics of cattle systems in low-to-middle-income tropical developing countries.

Within tropical regions, it is important to note the existence of geographic zones classified as temperate. Livestock located in these zones are typically raised on farmland characterized by plateaus or hillsides at altitudes equal to or higher than 1500 to 3000 m above sea level, where water is generally accessible year-round. Farm sizes in these areas often range from 200 to 2000 head of cattle, typically comprising European breeds (*Bos taurus*) such as Holstein or Jersey [23]. Also in some cases, crosses of specialized dairy cattle employ zero-grazing systems and sophisticated record-keeping software to manage daily events [17,20,21]. However, most other Latin American countries, including Costa Rica, Ecuador, and Colombia, use Holstein cattle to produce milk under year-round grazing conditions, with record keeping ranging from advanced digital software to modest paper-based systems. In contrast, livestock operations in lower-altitude tropical regions, between 600 and 1000 m, experience distinct climatic seasons, with dry and rainy months, which are an apparent constant issue directly impacting water availability and forage quantity, resulting in considerable variability in animal production [24].

Apart from geographical factors, the enterprise’s economic power to invest directly contributes to the farmer’s income. Live animals serve as a source of wealth and quick cash, whether sold for breeding or meat, while also supplying protein through meat or milk [25]. Tropical livestock production systems contribute between 2% and 24% of small farmers’ annual income [26] and play a crucial role in agricultural development, employment, and the livelihoods in tropical countries. Dairy and dual-purpose cattle in these regions play a significant role in alleviating poverty and hunger while improving human nutrition and economic development.

Additionally, the level of technology influences the unit’s productivity and performance. For example, Velázquez-Penagos et al. [27] classified farmers into those with high technological status, where farms implemented improved grazing systems and pastures, supplemented feed at the time of milking, administered annual preventive veterinary medicine (such as vaccinations and deworming), and frequent veterinary consultation concerning health, while keeping reproductive and productive records. These farmers often have higher production efficiencies. In contrast, farmers classified as medium-technology enterprises exhibited all the characteristics of the former but maintained rudimentary records, while the low-technology farmers fed their animals using native grazing pastures without supplementation, preventive medicine protocols, or record keeping. This study suggests that greater economic investment into the farm is associated with higher reproductive and productive outcomes.

**Table 1:** Key elements of cattle systems in low-to-middle-income tropical developing countries.

Animals [19,20,21,24]	*Bos indicus*, *Bos indicus* × *Bos taurus* crosses. High genetic diversity (not defined breeds). Breeds specialized for adaptability to hot and humid environments, not for productivity.
Nutrition Reproduction [24]	Native grasslands in extensive or semi-extensive conditions. Low nutritional supplementation. Mixed crops—low-quality forage. Seasonal variability of the crops.
Management [16,17,18,19,20,21,22,23,24,25,26,27]	Small-to-medium number of animals/farm (1–300 animals). Low veterinary consultation/input. Inconsistent or poor collection and interpretation of records. Low implementation of preventive veterinary medicine measures. Variable economic inputs and outputs. Traditional and outdated management and practices.
Technification [26,27]	Low adoption of technology. Low use of specialized cattle software and computer systems. Low-to-null use of agricultural precision systems. Lack of skilled professional personnel. Lack of performance data or computer software. Limited infrastructure with limited access to internet services.

These challenges can be partially ameliorated by collecting, keeping, and analyzing data and records. Dual-purpose systems have great potential in tropical areas and can become sustainably viable. However, due to low economic and technological investment coupled with an inadequate education and climatic variability, farmers face several limitations that make it difficult to optimize and improve the efficiency of the production system [20].

### 2.2. Importance of Record Keeping in Livestock Production Systems to Address Global Change

The collection of records at the farm level must be prioritized for optimal farm operations, and farmers must recognize the value of collecting information. This can be achieved by deriving economic profit from careful data analysis, thereby advising on intelligent decisions. In some places, decision making is based on market fluctuations in animal products; for instance, when there is a surplus of milk, farmers tend to produce fresh dairy products with a short shelf life. Similarly, if the demand for meat rises, farmers increase the sale of live animals, thereby jeopardizing herd renewal, especially if their heifer replacement program is poorly planned.

Depending on the type and content of the records, they can provide essential data for tracking trends, anticipating challenges, and implementing timely interventions [28,29,30]. This capability is particularly critical in adapting to the impacts of climate variability, such as shifts in disease patterns and marketing trends, which directly affect livestock health and production [31,32]. Additionally, record keeping facilitates the management of critical practices, including the implementation of feeding and breeding strategies and the mitigation of disease onset [33,34]. Moreover, record collection reinforces health programs by enabling real-time assessments of disease prevention strategies, such as vaccination schedules, parasite control, and biosecurity measures. This approach is particularly valuable, in regions vulnerable to vector-borne and infectious diseases exacerbated by climate change [35,36].

Accurate records foster collaboration with veterinary and agricultural experts and government officials, allowing for targeted, region-specific interventions [37,38,39]. Recently, traceability in livestock systems has enabled farmers to enter markets, allowing consumers access to essential information on the safety and quality of animal products and on the enterprise’s transparency and animal welfare [40,41].

Tropical livestock enterprises face several disadvantages and challenges associated with climatic variability that is less pronounced in temperate climates. This environmental variability, such as the high temperatures, humidity and the impacts on the forage quality and quantity, directly affects the animals’ characteristics (breeds, heterosis, adaptability, etc.) and their management, such as feeding strategies; herd health; economic, productive, reproductive aspects; and product marketing [42]. Studies from tropical countries highlight that consistent record keeping advances improves metrics. Unfortunately, the challenge of maintaining detailed records that could inform farmers about herd characteristics is particularly not evident in these regions, limiting their ability to manage emerging challenges effectively [43].

The development of data-recording software has enhanced herd monitoring, with programs measuring behavioral aspects such as activity levels, feed consumption, and ruminating and idle time. These data feed algorithms aim to diagnose several important aspects, such as sexual receptivity, health, and nutritional status. However, the implementation of these technologies remains limited in small tropical enterprises due to limited access to technology and the costs associated with purchasing software/devices and professional consulting services [44].

Introducing other technologies from temperate regions can also alter record-keeping practices. However, these often do not apply to tropical conditions and must be adapted. For example, artificial insemination has prompted farmers to revise records and to periodically monitor their animals more closely. The same cannot be said of embryo transfer, where local and international organizations sponsor most programs, with highly variable outcomes. Furthermore, when investment in this reproductive technology concludes, few studies examine the program’s benefits, thereby limiting its practicality for small-to-medium-sized farmers [45].

Records aim to improve the status and knowledge of tropical cattle operations. Efforts and investments should focus on information systems that are reliable and accessible for training smallholder farmer on software technologies. The implementation of decision-making processes is essential for improving productivity and animal welfare in these challenging tropical environments [46,47].

It is crucial to recognize that simpler record-keeping systems, such as in a calendar, are an easy way to introduce information, but a more complex way to analyze and interpret data. On the other hand, sophisticated software systems facilitate decision making but require constant updates, as more information must be incorporated into the system to be analyzed promptly and to enable timely interventions.

## 3. Records in Tropical Livestock Systems

### 3.1. Feasibility of Livestock Record-Keeping Systems in Low-to-Middle-Income Tropical Countries

Reports have shown that tropical livestock systems often lack even the most basic records compared with the detailed information used in temperate regions. In addition to mandatory identification records, most farmers will track only minimal data. Additionally, due to reproductive management and farm-level conditions, data on sires and their genetic backgrounds are rarely recorded and often missing [48,49].

Governments, international organizations, and researchers have invested considerable resources in modernizing tropical production systems, particularly by introducing digital record keeping. These initiatives aim to simplify animal management by providing accurate and dependable information. However, they have achieved limited success, especially in cattle raised under small farming conditions and in remote areas where internet access and electricity may be problematic [50].

In a review, Resti et al. [51] examined 24 electronic record-keeping systems used by dairy farmers in the tropics. This study divided data into four main groups: animal identification and registration, traceability, health information, and performance recording. It was observed that farmers are receptive to and willing to use technologies adapted to their specific conditions, such as low-to-intermittent internet connectivity, and prefer technologies that provide easy access to information via mobile devices. Unfortunately, there is no “one-size-fits-all” recording system that could alleviate this, as records are typically managed individually, with no system that encompasses all the information required for the different farm components, and these activities require time and effort from farmers.

**Figure 1:**
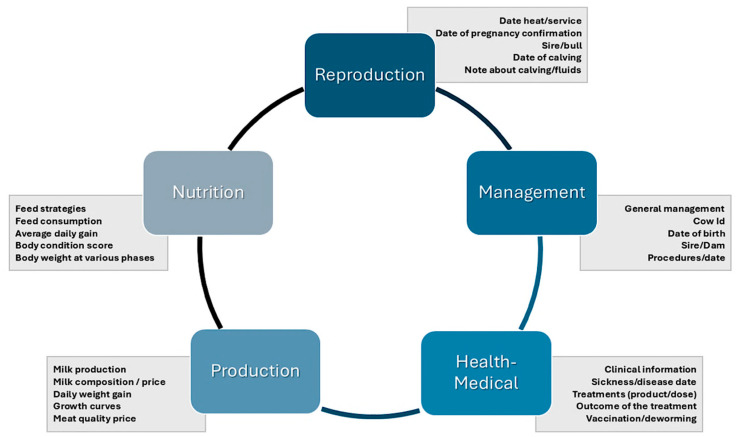
Proposed elementary domains required to collect, retain, and analyze data in animal production systems.

Figure 1 summarizes the proposed minimum information required to collect, retain, and analyze data, which can be categorized into five fundamental domains: general information about the cow and the farmer; production; reproduction; nutrition; and health. These minimum fields are a requirement helping farmers build a robust information system and address existing challenges in farm management.

### 3.2. Problems Found in Tropical Record Keeping: An Example of Reproductive Records

In the past, efforts to discern the value of information from the farmer’s records have been hindered by incomplete, inaccurate, or unreliable data weakening effective, productive and reproductive management strategies. As previously discussed, livestock in tropical systems exhibit lower reproductive performance than those in temperate climates [52,53]. The accuracy of the records is often limited by biases in the reporting data due to social, cultural and government pressures. Besides the language barriers, a lack of standardized knowledge and definitions of the different parameters contributes to the scarcity of information on reproductive parameters. Additionally, biased or incomplete reproductive records impede the ability of the farmers to optimize the biological (labor, animals, forage) and economical resources resulting in inefficient herd management.

Furthermore, the biological characteristics of the animals, such as growth phases, gestation lengths, lactation curves, and the cyclical reproductive physiology of open females, require time and effort to collect and maintain accurate data. Fertility traits in beef cattle raised under extensive conditions are complex and costly to measure and gather [54]. After collection, these records need precise, meaningful and timely analysis. This challenge is especially pronounced in extensive pastoral systems, where fertility-related information is scarce and often insufficiently detailed. For example, a Mexican study reported that 30% of records did not align with normal physiological parameters such as inaccurate gestation lengths, estrous cycles length and sexual receptivity intervals. Thus, the imprecise information of conception, calving and weaning dates makes the resulting data questionable [55].

Enríquez de la Fuente et al. [56] analyzed the reproductive records on paper and notecards to determine the best season for establishing a calving scheme in line with milk production. Their results showed that only 22% of the cows calved between 12 and 14 months, 24% between 15 and 17 months, and more than 50% calved after 17 months. However, the data only included cows that had successfully calved, excluding those that remained open or were culled. Galina and Arthur [52] reported a similar trend by calculating confidence intervals for 91 studies in a worldwide survey measuring calving intervals; the results indicated that for tropical cattle, the calving interval is around 15 to 16.1 months. Similarly, a more extensive survey of 263 studies measuring reproductive efficiency in the tropics reported a calving interval of 14–15 months [57].

Recently, Allan et al. [58] found that reproductive performance data in cattle from sub-Saharan countries are often limited by biased reporting, language barriers, and a lack of standardized definitions, all of which contribute to the scarcity of information on reproductive parameters. Similarly, biases were found in South African records, in which đ data outside a “suitable range” were omitted without explanation or specification of which animals were excluded from the study [59].

In this regard, the reliability of farm data is directly related to the time the farmer or administrator spends actively recording and registering the minimum data required, such as age at first calving, alongside calving and culling dates. Often, the input data contains errors and missing information. For instance, these inaccuracies are even more noticeable when farmers use artificial insemination (AI). Baca-Fuentes et al. [60] showed that in a farm routinely using this technique, 23% of the cows had a gestation length of approximately 11 months—far above normal physiological parameters—likely due to errors in pregnancy diagnosis or unrecorded natural mating.

Unfortunately, many studies on reproductive performance consider only animals that successfully reproduce. In Brazil, Balieiro et al. [61] analyzed 57,410 records for age at first calving in Nellore cattle from 1981 to 2002, aiming to calculate the genetic parameters for productive life and reproductive traits. Authors excluded records from cows with unknown paternity and those with only one date registered for calving, thereby introducing bias into the collected data. Only animals with age at first calving and calving intervals between 11 and 24 months were included in the analysis. This exclusion, while understandably based on the objectives of the study, only included females with successful pregnancies and not representing the entire nature of the population in the database. Simultaneously, in Benin, the average age at first calving was estimated at 37.2 months in Lagune cattle, ranging from 36 to 48 months [62]. However, the calving interval between calving and successful mating had not been determined due to the short duration of the study and the variety of mating practices, posing specific challenges for collecting demographic data within herds.

Another approach to determining reproductive parameters has been tested using structured questionnaires. For example, González-Padilla et al. [19] interviewed 3280 Mexican farmers, reporting that only about 50% maintain records and that fewer than 7% use computers for record keeping and analysis. The primary data recorded included reproductive information, calf management, and health practices. Interestingly, only 30% of the data were attributable to farm purchases and sales, which are closely linked to a farm’s turnover and economic measures.

Ilatsia et al. [63] asked farmers a simple question: Why does the Sahiwal breed perform better in southern Kenya than the East African Zebu? The opinion-based survey suggested that the latter breed is seen as more adaptable than the former. This example illustrates the trend of decision making based on views and hunches rather than previously analyzed data.

### 3.3. Adoption Rates of Record Keeping in Mixed Systems: Milk, Beef and Dual-Purpose

Traditionally, dual-purpose systems have minimal data collection and analysis. For instance, a study of dual-purpose systems in the lowland regions of Colombia found that only 43% of farmers kept records, and among those who did, some records were incomplete or contained inconsistencies that complicated data analysis [64]. Moreover, due to limited information from their farms, only 22% of farmers were aware of the costs associated with milk or meat production. Similarly, González-Quintero et al. [65] characterized 1313 farms, with 69.2% of Colombian herds classifying as small farms. The large farmers were more likely to maintain management records. In contrast, small farmers struggled to adopt technologies such as improved feeding, animal health, reproductive technologies, and data-based economic analysis. In the region, other studies have observed that only 9.7% of farmers keep records [66].

The lack of records significantly reduces animal efficiency and the economic profitability of the units [67]. For instance, the available information on records from these production systems typically comes from surveys and questionnaires administered at the farm level, and the data are primarily used for research purposes. Once the research or project is completed, farmers cease collecting data, likely due to a lack of incentives or an inability to appreciate its value [68].

Surveys and questionnaires have limitations in their implementation and in their long-term continuation. When farmers lack the culture or routine to collect and maintain records of farm events, establishing a baseline is a daunting undertaking that requires time and specialized personnel. This situation limits the analysis and understanding of farm circumstances and the dynamics of management events. Therefore, an important aspect of increasing productivity and decision making is fostering a culture of information collection that benefits farmers. This information could inform analysis and practical application, driving the necessary changes to increase the farm’s productivity and sustainability.

Whilst some overlap exists with the adoption of record keeping systems, the technology acceptance in production units in the lowland tropics can be influenced by various factors. Some of these include farm size, income, age and educational level of the farmer or manager. Furthermore, they include cultural and social predisposition to modifications, change in associated risks, and expectations regarding the direct profits. All these factors disturb record keeping and emphasize the difficulties when implementing technology/data collection at the farm level, in addition to time investment and resistance to changing traditional methods [50]. In this context, Salas-González et al. [69] assessed technology adoption among farmers in tropical regions of Mexico in response to a government livestock program. The study reveals that among the technology packages offered by the government, only 45.6% of farmers adopted record-keeping and general management tools. In comparison, adoption rates were 90.2% for pasture and range management, 81.3% for animal health and vaccination programs, and 65.4% for animal feeding.

A similar trend was observed in a study conducted in Ecuador, involving 41 small dual-purpose farms with fewer than 20 cows [70]. Adoption rates for decision-making and integrated management plans were only 28%, compared to other technological packages, such as animal health and feeding technologies (55%). This indicates that data collection is time-consuming and farmers do not recognize its true value and advantages, which could be reflected in economic benefits or improved management.

Moreover, when farmers were classified into economic input to the production units, low-income farmers adopted fewer technologies. Rangel et al. [17] evaluated and characterized 1475 farms in the Mexican tropics using socio-economic, geographic, and animal-oriented production parameters. Small-scale farmers exhibited low levels of technological adoption, including record keeping. It has been suggested that herds without records are less likely to improve their efficiency and sustainability [71]. Furthermore, implementing basic record keeping can have a strong and immediate impact at the farm level.

Most of the reports about record keeping come from Latin American countries; however, some data has also been generated in African countries such as Zimbabwe [72], Tanzania [73,74], Kenya [75] and South Africa [76]. For more information, please see Ragnekar and Thorpe [77], who reviewed the opportunities and constraints of African dairy production systems. Similarly to those from Latin America, African publications report a lack of proper collection of productive and reproductive records. Additionally, a review by McDermott et al. [78] highlights that dairy cows in the tropical regions of East Africa and South Asia can meet current protein demand and drive progress towards sustainable cattle production systems by enhancing management, health, feeding, and genetic merit. A critical aspect of these improvements is the collection of accurate and reliable data.

### 3.4. Challenges of Using Records in Livestock Production Systems in Low-to-Middle-Income Tropical Countries

Despite their potential to improve farm management and facilitate the decision-making process, record-keeping programs often face high dropout rates, as farmers discontinue their use due to a lack of interest or frustration with managing and interpreting the information. Several reasons have been proposed related to the inconsistency of attention using record-keeping programs:

1. The personnel managing the data may not be willing to spend time analyzing it; even if they do, they do not see immediate results.
2. Farmers are unable to update their records due to time constraints from other farm chores or participation in other economic activities [49].
3. The analysis could serve as a wake-up call to past poor decisions, making farm managers, owners, and other stakeholders uncomfortable about confronting these issues and the reality of the farm.
4. Cost and time associated with the collection and input of the information.
5. Adoption rates of record keeping ranges from 10 to 55% due to the lack of incentives, halted by human factors such as socio-cultural aspects, lack of resources and difficulties in implementation.

Some challenges related to the reluctance to adopt record-keeping systems include financial constraints, lack of technical support, risk aversion, a disconnect between the farm’s objectives and the implementation of the record-keeping system, plus the time and effort required for this activity. For example, the three top reasons for small farmers in Malawi for not keeping records are: 1. busy with other activities, 2. lack of knowledge, and 3. lack of education qualifications. The farmer’s level of education plays a direct role in the adoption and willingness to adopt a record-keeping system; only 39% of farmers attended elementary or senior primary education [79].

In some low-to-middle-income tropical countries, researchers have also observed that record keeping is linked to education, as high illiteracy and limited technical expertise prevent farmers from registering and interpreting data [80,81]. Many cannot see the benefits in the early stages of implementation and stop recording information. Therefore, tropical animal production systems must continue educating farmers, technical personnel, and administrators on the importance, use, analysis, and interpretation of record keeping.

The type of tools used to register events can also impact the adoption of effective record-keeping practices. Physical tools like cards or notebooks—while accessible and inexpensive—can be easily lost, torn, soiled, burned, or wet [82]. Additionally, further analysis of manually recorded data is often time-consuming and complex due to missing, unclear, or difficult-to-track information [83]. Mwanga et al. [84] suggested that small and medium-sized farmers from African countries do not keep or use handwritten records.

Digital records, although more accessible to analyze, have their drawbacks in the tropics, where basic infrastructure, such as reliable internet, computers, and training in digital systems/software, may be limited and economically infeasible. Financial constraints and limited awareness of available technologies obstruct many smallholders from the adoption of effective herd-management practices [85].

Additionally, recording tools may only sometimes meet the needs of cattle farmers. During the development of recording devices, the primary beneficiaries—farmers and cooperatives—should be more actively involved in this process, which is currently dominated by research institutions. Engaging and collaborating with relevant stakeholders in livestock production should help improve sustainable record keeping in low-to-middle-income tropical countries [51].

A study in Indonesia [80] showed that the best strategies for the improvement of smallholders on their production require farmers to focus on profitability and the use of proven management strategies including (a) using cattle breeds resistant/tolerant to environmental stressors, (b) understanding market preferences and behaviors, (c) managing cattle breeding herds based on rainfall patterns, (d) keeping good records on all aspects of breeding activities, and (e) adjusting stocking rates in extensive systems to match the grazing capacity of the land.

The lack of sound records is a constraint for production and farm management. It can also be a barrier to rigorous research in low-to-middle-income tropical countries, restricting policymakers from obtaining reliable information on the conditions of the livestock industry. Researchers often rely on direct observations or information provided by farmers, farm workers, extensionists, and cattlemen, using various techniques, including questionnaires, focus group discussions, and progeny history data collection [86,87,88].

Information is imprecise because many farmers rely on memory for everything from planting dates to input use. That can work for a while, but only accurate records will improve decisions over time. This situation directly influences the creation of custom, data-driven, and locally relevant solutions that can improve productive parameters and local economies in developing countries.

**Figure 2:**
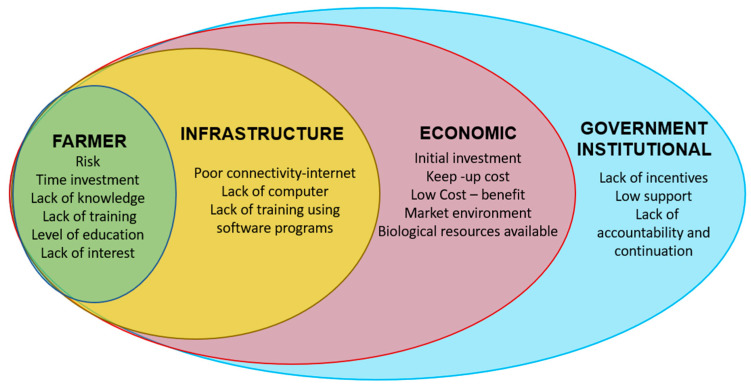
Four main barriers and challenges domains related to the adoption of record keeping programs in low-to-middle-income tropical farmers.

Figure 2 groups the barriers and challenges in four main domains related to the adoption of record keeping programs in low-to-middle-income tropical farmers.

### 3.5. Case Studies and Examples of Applied Record-Keeping in Tropical Livestock Systems

Explicit studies on the use of records are rare, and most research on record-keeping focuses on evaluating specific aspects of production, such as nutritional, reproductive, or management approaches. For example, in dual-purpose farms across four regions of Venezuela, a survey compared the productivity and finances of farmers who kept records with those who did not, revealing differences in overall productivity. It was demonstrated that farms with records generally performed better across productivity metrics, such as kilograms of milk or meat per pasture area [89]. Researchers observed substantial variation among farmers who kept records, ranging from 18% to 97%, depending on the type of record. Some farmers maintained economically productive records, whereas others preserved only accounting information. On average, farmers who kept accountable, productive records were more profitable than those who did not keep any or kept only one type of record.

The same pattern was observed in another Venezuelan study, in which milk and beef production efficiency was higher on farms that used records. Researchers also found that income composition differs between dual-purpose cattle farms with and without records: farms with records earned higher income from milk sales [90].

In other parts of the world, records show an improvement in farm efficiency. For instance, livestock recording information in Kenya is managed by three institutions that work with breeders and farmers to obtain baseline animal data, including pedigree and milk production records. Recording programs began in the 1970s, when farmers participated enthusiastically, but participation declined in subsequent years due to multiple factors [91]. Those who remain in the program use their information to decide and drive change within their production units.

In Indonesia, smallholder farmers recognized the importance of maintaining information on their enterprises, although not all practiced consistent record keeping [82]. These records were typically kept on cards to document cattle identification, genealogy, reproductive status, medical history, and growth data. Using records, 87% of farmers reported that livestock sales became more accessible, and that records also served as proof of animal ownership in the event of disputes. Furthermore, 67% of farmers agreed that the records facilitated access to health and reproductive services, enhancing the responsiveness of technicians providing livestock health services. The cards documented clinical histories, diseases, and treatments, thereby improving food safety and disease prevention [82].

Using farm records is also essential for developing breeding and genetic management programs, providing information on various performance indicators necessary for precise decision making, which is crucial when employing artificial insemination and embryo transfer [92].

In South Africa, the performance of five indigenous breeds was measured using pedigree records, which is crucial for preventing inbreeding and maintaining genetic diversity in the population [93]. Detailed and accurate records enable stakeholders to calculate estimated breeding values, evaluating multiple fertility traits, enhancing genetic improvement, thus establishing long-term breeding goals. Record evaluation enables breeders to identify which animals have superior fertility potential, ultimately leading to more productive and profitable cattle herds [4,54].

Some benefits associated with record keeping for farmers are (a) access to subsidies and government grants, for example, such as in the case of Kenyan farmers; (b) decreasing income variability risks; and (c) better-quality extension services, training, and resources from recording organizations and government agencies [91]. Record keeping is the source of information for the granting of financial and technical aid procured by the government. For instance, cattle farms in Turkey keeping physical or financial records are 89% more likely to receive agricultural support than their counterparts [94]. Farmers in Venezuela are also more likely to receive support or grants when they can provide evidence of their finances and operational efficiency [89].

In Thailand, as new technologies emerge, a mobile app was developed to educate and support farmers in record keeping [95]. This app was developed after interviewing small-scale farmers; although most did not keep records, all had smartphones with internet access, facilitating their participation in the program. The app is also informative, covering cow production cycles, general health, and feeding and calving management practices. Although apps can be promising, their performance depends on access to robust and well-maintained historical databases.

One example of a successful relationship between industry and academic institutions occurred in Costa Rica. Universidad Nacional in Costa Rica created VAMPP Bovino^®^ in conjunction with the University of Wageningen, Netherlands, and the Cooperativa de Productores de Leche Dos Pinos^®^, a dairy cooperative in Costa Rica. This liaison is an ex-cellent example of private industry participation in developing specialized milk produc-tion. This cooperative comprises almost 1500 associates from low- to mid-size dairy cattle farms. It has an average milking herd of 60 cows, thus incentivizing farmers to produce safe milk with optimal nutritional characteristics and to adhere to ethical practices. The University and the cooperative working together provide for their associates with comput-er equipment and the specialized software VAMPP Bovino^®^3.0, standardize information and support decision making.

The Universidad Nacional consolidates all VAMPP user databases—close to 2300 farms—into a national registry system [44,46]. This data can be used to report various strategies for improving milk production and genetics in dairy cattle in Costa Rica [96,97]. The trend indicates a positive effect of information system availability on productive and reproductive traits in the early years of implementation [44].

Another software widely used in Mexico and South America, particularly in Colombia, is Ganadero SG^®^. This package has been used to control and manage individual herds [98,99]. With a few exceptions, it has been used in population-level studies [100]. This program and VAMPP Bovino^®^ are the two most widely distributed in Latin America. However, several packages, such as Dairy Comp^®^, AgroVision^®^, and BoviSync^®^, are also used by farmers, particularly those with zero-grazing systems.

The use of specialized software to keep and analyze the records has several advantages as outlines previously; however, the main limitations and challenges of these systems are: 1. the cost associated with the initial purchase, implementation and maintenance of the equipment; 2. the time and cost associated with inputting the information into the software; 3. training and keeping updated personnel to work and manage the software; and 4. the reliability and accuracy of the systems to provide effective reports that represent a value for the farmer to drive decisions.

Table 2 summarizes typical and available documented papers reporting on records from tropical production systems. The papers are organized by country and present the key findings and how the records were used.

## 4. Future Directions

Since the 1990s, precision agricultural systems have revolutionized data collection, enabling precise and frequent monitoring of livestock. These advances have improved production indicators and reduced errors, emphasizing the global importance of record keeping in livestock systems. Despite these innovations, their application in tropical farms is limited. Gaps in infrastructure, unique environmental challenges, and a reluctance to adopt new technologies are the most common constraints. The lack of widespread adoption in tropical regions highlights the need for context-specific solutions.

To improve sustainability and productivity in tropical systems, future efforts must focus on developing affordable, robust tools, tailored to the specific needs of these regions, thus involving farmers and cooperatives in tool development [51]. It is imperative to provide farmers with training, resources, advisory and extension systems in order to show them the productive and economic benefits of record keeping, so they can adopt paper-based or digital technologies into their farm management practices [76].

Research should also be conducted to reduce the costs of digital record-keeping applications, so these technological applications are accessible to small-scale farmers, particularly those dedicated to livestock production [101]. Strengthening record-keeping practices in tropical systems is essential for optimizing resource use, achieving sustainability, addressing environmental challenges, and improving economic outcomes for farmers [37,102].

With the agronomic revolution and the use of agricultural precision systems, it is now possible to monitor animal performance in real time, allowing producers to continuously collect and analyze data from the farm [103]. Several authors have suggested the benefits of implementing precision systems, thus allowing for the facilitation of a cost-effective decision-making process based on data from the farm, providing the efficient use of biological resources (animals, land or water) and leading to increases in the economic return to the farmers [34,104,105]. The management of data and the assignment of personnel responsible for integrating the information (veterinarians or professional consultants) must be clearly defined and communicated to all parties [106].

In high-income countries, automated systems employing artificial intelligence and machine learning have recently increased in use, offering substantial advantages primarily for operational, medium- and long-term management [107,108,109,110]. Technologies have been developed outside the tropics and applied to extensive or pasture-based systems. Aquilani et al. [111] reviewed these technologies and their use to improve reproduction, nutrition, and prairie management; whether these technologies would be affordable to farmers in the tropics and adapted to local conditions remains to be tested.

Many farmers also own and use mobile phones—particularly smartphones—to engage in day-to-day activities. Integrating mobile technology improves data management, access to online veterinary services, and the availability of timely information, which farmers usually use to communicate crop and livestock costs and animal diseases [112]. Using mobile phone apps for record keeping offers new possibilities for data collection and analysis and creates an opportunity for farmers to use affordable technology.

In fact, Bernabucci et al. [113] suggest that the use of precision livestock systems within extensive systems is feasible if the system provides holistic integration of information, ranging from economic benefits to monitoring animal and pasture performance. However, the financial aspects of implementing these technologies on low-to-middle-income tropical farms remain largely unexplored. Finally, with the development and generation of artificial intelligence, there are opportunities for livestock systems to integrate these technologies not only with the analysis of the data collected at the farm, but to introduce innovations with the generation of new applications, such as deep learning with bio-sensors, the integration of management systems, facilitating decisions at the farm level, optimizing processes and increasing the productive and reproductive efficiency of livestock systems [114]. One important aspect to consider with artificial intelligence and deep learning technologies, which could be implemented in farms classified as low-to-middle systems, is that they have to be highly adaptable, flexible and economically affordable to the farmers. In fact, if artificial intelligence and deep learning systems are distributed uniformly across countries and livestock systems, disparities will occur. However, these technologies have the potential to revolutionize methods and processes at the farm level, and they will become more affordable and adaptable to the tropical conditions with time.

## 5. Conclusions

Effective record keeping is central to improving sustainable livestock productivity and economic outcomes. Accurate data collection and analysis allow farmers to make better decisions regarding feeding, reproductive, health management, and environmental stewardship, thereby promoting more efficient utilization of the land, preserving soil health and biodiversity, and advancing sustainability. Accurate, detailed records enable farmers to monitor key farm components, such as milk yield, reproductive health, and disease incidence, therefore facilitating better decision making and optimizing resource use. These records also help with product traceability, ensuring safety and quality while building consumer trust and improving access to premium markets.

Many barriers prevent farmers from using records, but several solutions have been identified to address low adoption rates. With the reservations of our sources of information, the three proposed causes of low adoption are:

1. Educating and delivering training programs to farmers is crucial for improving the adoption of record-keeping practices.
2. Programs must engage farmers and other stakeholders in developing tools, prioritizing the needs of tropical low-to-middle-income systems and the unique characteristics of cattle in the region.
3. Implementing strategies to educate farmers, stakeholders, and technical personnel in the use, analysis, and the interpretation of information is essential to creating a robust system for reliable data collection and storage.

These issues must be prioritized for the future sustainability of farming in the low-to-middle-income livestock tropical systems. Finally, as described in this review, the low adoption, collection and interpretation of records are attributed to many causes. However, with the development of new technologies and production systems, the future of animal production is promising to become efficient and sustainable and to alleviate the hunger of the growing population of the world, meeting society needs.

## Figures and Tables

**Table 2:** Information on the use of records and key findings in tropical livestock systems.

Country	Study	Key Findings	Use of Records
Benin	Ahozonlin & Dossa, [62]	Productive and reproductive performance of Lagune cattle under village conditions, showing an average age at first calving of 37.2 months and variability in calving intervals.	Reproductive records were scarce, and calving intervals could not be determined due to insufficiently consistent data.
Brazil	Baliero et al. [61]	Genetic parameters for productive life and reproductive efficiency in Nellore cattle, highlighting the importance of records for genetic improvement.	Productive and reproductive records were essential for calculating genetic values and improving reproductive efficiency in Nellore cattle.
Colombia	Cortés Mora et al. [64]	Characterization of dual-purpose production systems in the humid tropics, highlighting the lack of records and the need to improve production efficiency.	Only 43% of farmers kept incomplete or inconsistent records, making it challenging to analyze the information.
Colombia	González-Quintero et al. [65]	Technical and environmental analysis of dual-purpose farms, identifying ways to improve production and sustainability.	Medium and large farmers were more likely to maintain management records, while small farmers struggled to implement record-keeping systems.
Costa Rica	Martínez et al. [96]	Comparison of the productive performance of cows born through embryo transfer, artificial insemination, and natural mating in dairy and dual-purpose herds under tropical conditions.	Records were used to compare cows’ productive and reproductive performance under different reproduction methods.
Costa Rica	Vargas-Leitón et al. [97]	Comparison of lifetime milk production in Holstein, Holstein–Gyr, and Holstein–Brahman crossbred cows under tropical conditions.	Production and reproductive records were essential for evaluating the long-term performance of cows across different crossbreeding systems.
Costa Rica	Sánchez-Hernández et al. [44]	Use of the dairy cattle software to support cattle population research and improve productivity in dairy herds.	The software was used to standardize information and support decision making, with 1500 associates using the system.
Costa Rica	Romero-Zúñiga et al. [46]	Implementation of a software as a tool for dialog between the bovine production sector and academia.	The software enabled the recording of individual herd data, which was consolidated into a national registry system for research.
Costa Rica	Baca Fuentes et al. [60]	Reproductive behavior of *Bos taurus* × *Bos indicus* heifers artificially inseminated in the dry tropics of Costa Rica.	Records showed that 23% of cows had gestation lengths of around 11 months, likely due to inaccuracies in pregnancy diagnosis or breeding dates.
Ecuador	Torres et al. [70]	Identification and implementation of technological packages for dual-purpose cattle in Manabí, highlighting the low adoption of records and the need for training.	Only 28% of farmers used records in decision making, thereby limiting the implementation of integrated management plans.
Honduras	Copas Medina et al. [100]	Effect of age at first calving on longevity and milk production in Holstein and Brown Swiss cows, highlighting the importance of records for decision making.	Production and reproductive records were used to evaluate the impact of age at first calving on longevity and milk production.
India	Rangnekar & Thorpe, [77]	Opportunities and limitations in smallholder dairy production and marketing, highlighting the need to improve management and market access.	The lack of records was identified as a key limitation for improving productivity and marketing in smallholder systems.
Indonesia	Abdullah & Mustabi, [82]	Benefits of cattle recording cards in beef cattle breeding, facilitating the sale of animals and access to health and reproductive services.	Recording cards helped farmers document identification, genealogy, reproductive status, and medical history, improving management and marketing.
Kenya	Wasike et al. [91]	Factors influencing the efficiency of cattle recording systems in Kenya, identifying opportunities and threats for the adoption of records.	Farmers who used records recognized improvements in decision making and access to premium markets, but adoption was limited due to resistance to change.
Kenya	Ilatsia et al. [63]	Production objectives and breeding goals of Sahiwal cattle keepers in Kenya, highlighting the importance of adaptability and reproductive performance.	Reproductive records were scarce, and farmers made decisions based on perceptions rather than recorded data.
Malawi	Chagunda et al. [79]	Analysis of smallholder farmers’ willingness to adopt dairy performance recording systems and identification of barriers.	Farmers did not maintain written records due to limited knowledge, time, and education, thereby limiting the adoption of record-keeping systems.
Mexico	Velázquez-Penagos et al. [27]	Effect of technological status on the induction of ovarian activity in dual-purpose cattle under tropical conditions.	Reproductive records were used to evaluate the efficiency of inducing ovarian activity in herds with different technological levels.
Mexico	Salas-González et al. [69]	Technology adoption among farmers in tropical regions of Mexico, focusing on record-keeping and general management tools.	Only 45.6% of farmers adopted record-keeping tools, compared with higher adoption rates for other technologies, such as pasture management (90.2%) and animal health (81.3%).
Mexico	Rangel et al. [17]	Structural and technological characterization of smallholder dual-purpose cattle farms in Mexico.	Small-scale farmers exhibited low levels of technological adoption, including record keeping, due to financial constraints and a lack of technical support.
Mexico	Magaña-Monforte et al. [71]	Challenges of dual-purpose systems in tropical climates in Mexico, highlighting the need for improved management and record keeping.	The lack of records was a significant limitation for improving efficiency and sustainability in dual-purpose systems.
South Africa	Maciel et al. [59]	Factors influencing Nguni cattle’s reproductive and productive performance highlight the importance of management and data recording.	Reproductive records were used to evaluate cow performance; however, some data were excluded because they were out of range, which affected the analysis’s accuracy.
South Africa	Nkadimeng et al. [76]	Establishment of benchmarks for smallholder beef cattle herds in South Africa, focusing on reproductive performance.	Reproductive records were essential for establishing benchmarks and evaluating herd performance, though adoption among smallholders was limited.
Tanzania	Munyaneza et al. [74]	Identifying sustainability indicators in smallholder dairy production systems highlights the importance of management and data recording.	The lack of consistent records was identified as a barrier to evaluating sustainability and improving productivity.
Tanzania	Maleko et al. [73]	Analysis of feeding technologies in smallholder dairy farmers, identifying failures, successes, and challenges for sustainability.	Feeding and production records were scarce, making it difficult to assess the impact of feeding technologies.
Thailand	Saengwong et al. [95]	Development of a mobile app for recording and managing alerts on smallholder beef cattle farms, facilitating the adoption of digital technologies.	The mobile app allowed farmers to record and manage production, health, and reproductive data, improving decision making and efficiency.
Uruguay	Naya et al. [54]	Modeling fertility traits in beef cattle using linear and non-linear models highlights the complexity and cost of measuring these traits in extensive systems.	Fertility records were difficult to obtain in extensive systems, limiting the ability to analyze and improve genetic performance.
Venezuela	Padrón Morales et al. [89]	The impact of accounting and production records on the profitability of dual-purpose farms shows that farms with records were more profitable.	Farms that maintained accounting and production records showed higher income from milk sales and better profitability than those that did not.
Venezuela	Silva et al. [90]	Use of control records and performance indicators in dual-purpose cattle farming, highlighting the importance of records for improving efficiency.	Control records permitted farmers to monitor key indicators, such as milk production and reproductive efficiency, thereby improving decision making.
Zimbabwe	Tavirimirwa et al. [72]	Review of cattle production in communal systems, highlighting challenges and opportunities to improve productivity and sustainability.	The lack of records was a key challenge in communal systems, limiting farmers’ ability to monitor and improve productivity.

## Data Availability

Data sharing is not applicable to this article.

## References

[1] United Nations (2024). Population.

[2] World Bank (2024). World Development Report 2024: Economic Growth in Middle-Income Countries. https://www.worldbank.org/en/publication/wdr2024.

[3] Kowalski A.M., Filho W.L., Azul A.M., Brandli L., Salvia A.L., Özuyar P.G., Wall T. (2021). Global South-Global North Differences. Encyclopedia of the UN Sustainable Development Goals.

[4] Marchioretto P.V., Rabel R.A.C., Allen C.A., Ole-Neselle M.M.B., Wheeler M.B. (2023). Development of genetically improved tropical-adapted dairy cattle. Anim. Front..

[5] Herrero M., Grace D., Njuki J., Johnson N., Enahoro D., Silvestri S., Rufino M.C. (2013). The roles of livestock in developing countries. Animal.

[6] Molina-Benavides R.A., Perilla-Duque S.M., Campos-Gaona R., Sánchez-Guerrero H., Rivera-Palacio J.C., Muñoz-Borja L.A., Jiménez-Rodas D.R. (2023). Effect of climate on thermal response in cows of different racial groups in lower tropic. Rev. MVZ Córdoba.

[7] Edvan R.L., Rocha-Bezerra L., Torreao-Marques C.A. (2015). Shortage of Biodiversity in Grassland. Biodiversity in Ecosystems—Linking Structure and Function.

[8] Madhusudan M.D. (2004). Recovery of wild large herbivores following livestock decline in a tropical Indian wildlife reserve. J. Appl. Ecol..

[9] Thornton P.K., van de Steeg J., Notenbaert A., Herrero M. (2009). The impacts of climate change on livestock and livestock systems in developing countries: A review of what we know and what we need to know. Agric. Syst..

[10] Tyukavina A., Hansen M.C., Potapov P.V., Stehman S.V., Smith-Rodriguez K., Okpa C., Aguilar R. (2017). Types and rates of forest disturbance in Brazilian Legal Amazon, 2000–2013. Sci. Adv..

[11] Manzano P., del Prado A., Pardo G. (2023). Comparable GHG emissions from animals in wildlife and livestock-dominated savannas. npj Clim. Atmos. Sci..

[12] Hsiang S.M., Meng K.C. (2015). Tropical Economics. Am. Econ. Rev..

[13] dos Santos J.S., Miziara F., Fernandes H.d.S., Miranda R.C., Collevatti R.G. (2021). Technification in Dairy Farms May Reconcile Habitat Conservation in a Brazilian Savanna Region. Sustainability.

[14] Latawiec A.E., Strassburg B.N.B., Silva D., Alves-Pinto H.N., Feltran-Barbieri R., Castro A., Iribarrem A., Cordeiro Rangel M., Kalif K.A.B., Gardner T. (2017). Improving land management in Brazil: A perspective from producers. Agric. Ecosyst. Environ..

[15] Herrero M., Thornton P.K., Gerber P., Reid R.S. (2009). Livestock, livelihoods and the environment: Understanding the trade-offs. Curr. Opin. Environ. Sustain..

[16] Seré C., Steinfeld H., Groenewold J. (1996). World Livestock Production Systems.

[17] Rangel J., Perea J., De-Pablos-Heredero C., Espinosa-García J.A., Mujica P.T., Feijoo M., Barba C., García A. (2020). Structural and Technological Characterization of Tropical Smallholder Farms of Dual-Purpose Cattle in Mexico. Animals.

[18] Romanzini E.P., Barbero R.P., Reis R.A., Hadley D., Malheiros E.B. (2020). Economic evaluation from beef cattle production industry with intensification in Brazil’s tropical pastures. Trop. Anim. Health Prod..

[19] González-Padilla E., Lassala A., Pedernera M., Gutiérrez C.G. (2019). Cow-calf management practices in Mexico: Farm organization and infrastructure. Vet. Mex. OA.

[20] Rojo-Rubio R., Vázquez-Armijo J.F., Pérez-Hernández P., Mendoza-Martínez G.D., Salem A.Z.M., Albarrán-Portillo B., González-Reyna A., Hernández-Martínez J., Rebollar-Rebollar S., Cardoso-Jiménez D. (2009). Dual purpose cattle production in Mexico. Trop. Anim. Health Prod..

[21] Hernandez A., Galina C.S., Geffroy M., Jung J., Westin R., Berg C. (2022). Cattle welfare aspects of production systems in the tropics. Anim. Prod. Sci..

[22] Uzcátegui-Varela J.P., Chompre K., Castillo D., Rangel S., Briceño-Rangel A., Piña A. (2022). Nutritional assessment of tropical pastures as a sustainability strategy in dual-purpose cattle ranching in the South of Lake Maracaibo, Venezuela. J. Saudi Soc. Agric. Sci..

[23] Durana C., Murgueitio E., Murgueitio B. (2023). Sustainability of dairy farming in Colombia’s High Andean region. Front. Sustain. Food Syst..

[24] Fariña S.R., Baudracco J., Bargo F. (2020). Dairy production in diverse regions: Latin America. Encycl. Dairy Sci..

[25] Banda L.J., Tanganyika J. (2021). Livestock provide more than food in smallholder production systems of developing countries. Anim. Front..

[26] Pica-Ciamarra U., Tasciotti L., Otte J., Zezza A. Livestock Assets, Livestock Income and Rural Households: Cross-Country Evidence from Household Surveys. ESA Working Papers, Food and Agriculture Organization of the United Nations, Agricultural Development Economics Division (ESA) 2011, Article 289004. https://ideas.repec.org//p/ags/faoaes/289004.html.

[27] Velázquez-Penagos H., Galindo-Rodríguez L., Barrientos-Morales M., Galina C.S., Maquivar M., Montiel-Palacios F. (2020). Effect of the Technological Status of Small Cow-Calf Farm Producers on the Induction to Resumption of Ovarian Activity of Dual-Purpose Cattle Raised under Topical Conditions. Open J. Vet. Med..

[28] Garcia S.N., Osburn B.I., Cullor J.S. (2019). A one health perspective on dairy production and dairy food safety. One Health.

[29] Garcia S.N., Osburn B.I., Jay-Russell M.T. (2020). One Health for Food Safety, Food Security, and Sustainable Food Production. Front. Sustain. Food Syst..

[30] Gerber P.J., Steinfeld H., Henderson B., Mottet A., Opio C., Dijkman J., Falcucci A., Tempio G. (2013). Tackling Climate Change Through Livestock—A Global Assessment of Emissions and Mitigation Opportunities.

[31] FAO (2020). Animal Health and Climate Change: Protecting the Health of Animals to Help Reduce the Effects of Our Changing Climate on Hunger and Poverty. https://openknowledge.fao.org/server/api/core/bitstreams/da3f8543-c638-4d98-9e23-d5a0db708ff3/content.

[32] Nejash A. (2016). Impact of Climate Change on Livestock Health: A Review. J. Biol. Agric. Healthc..

[33] Opio C. (2020). Livestock Under Climatechange Adaptation of Livestock Systems to Climate Change.

[34] Papakonstantinou G.I., Voulgarakis N., Terzidou G., Fotos L., Giamouri E., Papatsiros V.G. (2024). Precision Livestock Farming Technology: Applications and Challenges of Animal Welfare and Climate Change. Agriculture.

[35] Chang Q., Zhou H., Khan N., Ma J. (2023). Can Climate Change Increase the Spread of Animal Diseases? Evidence from 278 Villages in China. Atmosphere.

[36] Gale P., Drew T., Phipps L.P., David G., Wooldridge M. (2009). The effect of climate change on the occurrence and prevalence of livestock diseases in Great Britain: A review. J. Appl. Microbiol..

[37] Bach A., Ahedo J. (2008). Record Keeping and Economics of Dairy Heifers. Vet. Clin. N. Am. Food Anim. Pract..

[38] Brand A., Noordhuizen J.P.T.M., Schukken Y.H. (1996). Herd Health and Production Management in Dairy Practice.

[39] Lacetera N. (2019). Impact of climate change on animal health and welfare. Anim. Front..

[40] Leroy G., Fernando M., FAO (2019). Developing sustainable value chains for small-scale producers. Animal Production and Health Guidelines, 21.

[41] FarmFit Insights Hub (2024). Farm Management Information Systems (FMIS) Guide: Transforming Smallholder Agricultural Markets. Farmfit Playbook. https://farmfitinsightshub.org/resources/farm-management-information-systems-fmis.

[42] Galina C.S., Rubio I. (2001). New Perspectives and Opportunities for Improving Reproduction in Dual Purpose Cattle.

[43] Bett B., Kiunga P., Gachohi J., Sindato C., Mbotha D., Robinson T., Lindahl J., Grace D. (2017). Effects of climate change on the occurrence and distribution of livestock diseases. Prev. Vet. Med..

[44] Sánchez-Hernández Z., Galina-Hidalgo C.S., Vargas-Leitón B., Rojas-Campos J., Estrada-König S. (2020). Herd management information systems to support cattle population research: The VAMPP^®^ case. Agron. Mesoam..

[45] Contreras D.A., Galina C.S., Chenoweth P. (2021). Prospects for increasing the utilization of cattle embryo transfer by small-scale milk and meat producers in tropical regions. Reprod. Domest. Anim..

[46] Romero-Zúñiga J.J., Rojas Campos J., Bolaños Segura M., Castillo-Badilla G., Vargas-Leitón B., König S.E. (2019). Software Vampp Bovino como instrumento de mediación dialógica entre el sector productivo bovino y la academia. Univ. En. Diálogo Rev. De Extensión.

[47] Sánchez Z., Galina C.S., Vargas B., Romero J.J., Estrada S. (2020). The Use of Computer Records: A Tool to Increase Productivity in Dairy Herds. Animals.

[48] Hooven N.W. (1978). Cow Identification and Recording Systems. J. Dairy Sci..

[49] Yadeta W., Habte D., Kassa N., Befekadu B., Fetene E. (2020). Dairy Farm Record Keeping with Emphasis on its Importance, Methods, Types, and Status in Some Countries. Int. J. Res. Stud. Biosci..

[50] Galina C.S., Turnbull F., Noguez-Ortiz A. (2016). Factors Affecting Technology Adoption in Small Community Farmers in Relation to Reproductive Events in Tropical Cattle Raised under Dual Purpose Systems. Open J. Vet. Med..

[51] Resti Y., Reynoso G.G., Probst L., Indriasari S., Mindara G.P., Hakim A., Wurzinger M. (2024). A review of on-farm recording tools for smallholder dairy farming in developing countries. Trop. Anim. Health Prod..

[52] Galina C.S., Arthur G.H. (1989). Review of Cattle Reproduction in the Tropics: Parturition and Calving Intervals. Anim. Breed. Abstr..

[53] Maquivar M., Galina C. (2010). Factors Affecting the Readiness and Preparation of Replacement Heifers in Tropical Breeding Environments. Reprod. Domest. Anim..

[54] Naya H., Peñagaricano F., Urioste J.I. (2017). Modelling female fertility traits in beef cattle using linear and non-linear models. J. Anim. Breed. Genet..

[55] Fuentes M.C., Galina C.S., Navarro-Fierro R. (1988). Reliability of reproductive records for the study of reproductive efficiency in the tropics. Proceedings of the 11th International Congress of Animal Reproduction and Artificial Insemination.

[56] Enríquez de la Fuente B.A., Galina Hidalgo C.S., Navarro R., Gutierrez A.C. (1993). Estimación de la época más propicia para un empadre estacional en ganado cebú bajo condiciones de trópico húmedo. Av. Investig. Agropecu..

[57] Anta E., Rivera J.A., Galina C., Porras A., Zarco-Quintero L. (1989). An analysis of the information published in Mexico in relation to the reproductive efficiency of the bovine. II Reproductive parameters. Vet. Mex..

[58] Allan F.K., MacVicar I.S., Peters A.R., Schnier C. (2024). Systematic map of recent evidence on reproductive performance of cattle in Africa. Trop. Anim. Health Prod..

[59] Maciel S.M.A., Fair M.D., Scholtz M.M., Neser F.W.C. (2016). Factors influencing the reproduction and production performance of the Nguni cattle ecotypes in South Africa. Trop. Anim. Health Prod..

[60] Baca Fuentes J.R., Pérez Gutiérrez E., Galina-Hidalgo C.S. (1998). Comportamiento reproductivo de novillas Bos taurus x Bos indicus inseminadas artificalmente a estro natural en el trópico seco de Costa Rica. Vet. Méx.

[61] Balieiro J.C.C., Eler J.P., Ferraz J.B.S., Mattos E.C., Balieiro C.C. (2008). Genetic parameters for productive life traits and reproductive efficiency traits at 6 years in Nellore cattle. Genet. Mol. Res..

[62] Ahozonlin M.C., Dossa L.H. (2022). Productive and reproductive performances of smallholder West African shorthorn Lagune cattle herds under village conditions in Southern Benin. Trop. Anim. Health Prod..

[63] Ilatsia E.D., Roessler R., Kahi A.K., Piepho H.-P., Zárate V. (2012). Production objectives and breeding goals of Sahiwal cattle keepers in Kenya and implications for a breeding programme. Trop. Anim. Health Prod..

[64] Cortés Mora J.A., Cotes Torres A., Cotes Torres J.M. (2012). Características estructurales del sistema de producción con bovinos doble propósito en el trópico húmedo colombiano. Rev. Colomb. Cienc. Pecu..

[65] González-Quintero R., Barahona-Rosales R., Bolívar-Vergara D.M., Chirinda N., Arango J., Pantévez H.A., Correa-Londoño G., Sánchez-Pinzón M.S. (2020). Technical and environmental characterization of dual-purpose cattle farms and ways of improving production: A case study in Colombia. Pastoralism.

[66] Burgos-Paz W., Pérez-Escobar Y., Castillo Losada E., Rivera-Sanchez L., Falla-Tapias S. (2025). Evaluating the Breed and Production Diversity in Dual Purpose Cattle Systems in Colombia: Opportunities for Its Sustainability. Agriculture.

[67] Castillo A.D., López Y.S., Corría E.C., Corrales C.P., Vázquez H.J., Zubiaur R.O.M., Vázquez T.E.R., Sánchez M.F.D., Cruz A.F.M., Cruz O.G. (2014). Caracterización de ranchos ganaderos de Campeche, México. Resultados de proyectos de transferencia de tecnologías. Av. Investig. Agropecu..

[68] Anderson S., Santos J., Boden R., Wadsworth J. (1992). Characterization of cattle production systems in the state of Yucatan. Proceedings of the Dual Purpose Cattle Production Research.

[69] Salas González J.M., Leos Rodríguez J.A., Sagarnaga Villegas L.M., Zavala-Pineda M.J. (2013). Adopción de tecnologías por productores beneficiarios del programa de estímulos a la productividad ganadera (PROGAN) en México. Rev. Mex. De Cienc. Pecu..

[70] Torres Y., Rivas J., Pablos-Heredero D., Perea J., Toro-Mujica P., Angon E., Garcia A. (2014). Identification and implementation of technological packages for dual purpose cattle: A case study of Manabí-Ecuador. Rev. Mex. De Cienc. Pecu..

[71] Magaña-Monforte J.G., Rios-Arjona G., Martínez-González J. (2006). Dual purpose cattle production systems and the challenges of the tropics of Mexico. Arch. Latinoam. Prod. Anim..

[72] Tavirimirwa B., Mwembe R., Ngulube B., Banana N., Nyamushamba G., Ncube S., Nkomboni D. (2013). Communal cattle production in Zimbabwe: A review. Livest. Res. Rural Dev..

[73] Maleko D., Msalya G., Mwilawa A., Pasape L., Mtei K. (2018). Smallholder dairy cattle feeding technologies and practices in Tanzania: Failures, successes, challenges and prospects for sustainability. Int. J. Agric. Sustain..

[74] Munyaneza C., Kurwijila L.R., Mdoe N.S., Baltenweck I., Twine E.E. (2019). Identification of appropriate indicators for assessing sustainability of small-holder milk production systems in Tanzania. Sustain. Prod. Consum..

[75] Odero-Waitituh J. (2017). Smallholder dairy production in Kenya: A review. Livest. Res. Rural Dev..

[76] Nkadimeng M., Van Marle-Köster E., Nengovhela N.B., Ramukhithi F.V., Mphaphathi M.L., Rust J.M., Makgahlela M.L. (2022). Assessing Reproductive Performance to Establish Benchmarks for Small-Holder Beef Cattle Herds in South Africa. Animals.

[77] Rangnekar D., Thorpe W. Smallholder dairy production and marketing—Opportunities and constraints. Proceedings of the South-South Workshop held at National Dairy Development Board (NDDB).

[78] McDermott J.J., Staal S.J., Freeman H.A., Herrero M., Van de Steeg J.A. (2010). Sustaining intensification of smallholder livestock systems in the tropics. Livest. Sci..

[79] Chagunda M., Msiska A., Wollny C., Tchale H., Banda J. (2006). An analysis of smallholder farmers’ willingness to adopt dairy performance recording in Malawi. Livest. Res. Rural Dev..

[80] Burrow H. (2019). Strategies for Increasing Beef Cattle Production under Dryland Farming Systems. Wartazoa.

[81] Prajapati M.R., Vahoniya D., Lad Y. (2020). A study on status of farm record keeping practices among the farmers in Anand Taluka. Int. J. Bus. Gen. Manag..

[82] Abdullah A., Mustabi J. (2020). Analysis of the benefits of cattle recording cards in beef cattle breeding from the perspective of farmers. IOP Conf. Ser. Earth Environ. Sci..

[83] Basir M.S., Buckmaster D., Raturi A., Zhang Y. (2024). From pen and paper to digital precision: A comprehensive review of on-farm recordkeeping. Precis. Agric..

[84] Mwanga G., Mbega E., Yonah Z., Chagunda M.G.G. (2020). How Information Communication Technology Can Enhance Evidence-Based Decisions and Farm-to-Fork Animal Traceability for Livestock Farmers. Sci. World J..

[85] Duval J.E., Bareille N., Madouasse A., De Joybert M., Sjöström K., Emanuelson U., Bonnet-Beaugrand F., Fourichon C. (2018). Evaluation of the impact of a Herd Health and Production Management programme in organic dairy cattle farms: A process evaluation approach. Animal.

[86] Iles K. (1994). The progeny history data collection technique: A case study from Samburu District, Kenya. RRA Notes.

[87] Amarasekera S. (1998). Animal Recording in Smallholder Farming Systems. The Sri Lankan Experience.

[88] Siegmund-Schultze M., Lange F., Schneiderat U., Steinbach J. (2012). Performance, management and objectives of cattle farming on communal ranges in Namibia. J. Arid Environ..

[89] Padrón Morales S.M., Velasco J., Urdaneta F. (2012). Los registros contables y productivos y su interacción con los resultados económicos en fincas ganaderas de doble propósito del estado Zulia. Rev. Fac. Agron..

[90] Silva D., Peña M.E., Urdaneta F. (2010). Registros de control e indicadores de resultados en ganadería bovina de doble propósito. Rev. Científica.

[91] Wasike C.B., Magothe T.M., Kahi A.K., Peters K.J. (2011). Factors that influence the efficiency of beef and dairy cattle recording system in Kenya: A SWOT–AHP analysis. Trop. Anim. Health Prod..

[92] Van Arendonk J.A.M. (2011). The role of reproductive technologies in breeding schemes for livestock populations in developing countries. Livest. Sci..

[93] Abin S.A.M. (2014). Animal Recording as a Tool for Improved Genetic Management in African Beef Cattle Breeds. Master’s Thesis.

[94] Alhas Eroglu N., Bozoglu M., Bilgic A. (2020). The Impact of Livestock Supports on Production and Income of the Beef Cattle Farms: A Case of Samsun Province, Turkey. J. Agric. Sci..

[95] Saengwong S., Intawicha P., Phuwisaranakom P. (2021). Assisting Knowledge Dissemination of Postpartum Beef Cows Management using Smartphone-Based Technology. Walailak J. Sci. Tech..

[96] Martínez J.F., Galina C.S., Rojas J., Vargas B., Romero-Zúñiga J.J. (2023). Comparative productive performance of cows born through embryo transfer, artificial insemination and natural mating in dairy and dual-purpose herds raised in tropical conditions. Reprod. Domest. Anim..

[97] Vargas-Leitón B., Romero-Zúñiga J.J., Rojas J., Galina C.S., Martínez J.F. (2024). Lifetime milk production of Holstein cattle in the humid tropics compared to Holstein-Gyr and Holstein-Brahman crosses. Reprod. Domest. Anim..

[98] Ayala Parra J.A. (2015). Implementación de un Software Ganadero sg en la Hacienda Santa Barbara (Municipio de Pinchote-Santander). Ph.D. Thesis.

[99] Taborda Pérez N. (2019). Análisis de Datos del Software Ganadero en Ganados Monterrey S.A. Obteniendo Parámetros Productivos y Reproductivos para Generar una Proyección Competitiva y Eficiente en la Empresa. Ph.D. Thesis.

[100] Copas Medina K.A., Valladares Rodas M., Baeza Rodríguez J.J., Magaña Monforte J.G., Segura Correa J.C., Copas Medina K.A., Valladares Rodas M., Baeza Rodríguez J.J., Magaña Monforte J.G., Segura Correa J.C. (2022). Efecto de la edad al primer parto sobre la longevidad, el número de días en producción y la producción de leche durante la vida productiva de las vacas lecheras Holstein y Pardo Suizo en Honduras. Rev. Mex. De cienc. Pecu..

[101] Ariza-Colpas P., Morales-Ortega R., Piñeres-Melo M.A., Melendez-Pertuz F., Serrano-Torné G., Hernandez-Sanchez G., Martínez-Osorio H., Collazos-Morales C., Figueroa-García J.C., Duarte-González M., Jaramillo-Isaza S., Orjuela-Cañon A.D., Díaz-Gutierrez Y. (2019). Teleagro: Software Architecture of Georeferencing and Detection of Heat of Cattle. Applied Computer Sciences in Engineering.

[102] International Committee for Animal Recording (ICAR) (2024). Historic Information About Milk Recording. The Global Standard for Livestock Data. https://www.icar.org/index.php/about-us-icar-facts/historic-information-about-milk-recording/.

[103] Nsabiyeze A., Zhang M., Li J., Zhao Q., Zhang X. (2025). Precision livestock farming for climate-resilient livestock management: A review of real-time monitoring and decision support systems. J. Clean. Prod..

[104] Jiang B., Tang W., Cui L., Deng X. (2023). Precision Livestock Farming Research: A Global Scientometric Review. Animals.

[105] Horváthné Kovács B., Zörög Z. (2025). Digital livestock farming in climate-smart agriculture: An overview to advance the SDGs. Discov. Sustain..

[106] Kleen J.L., Guatteo R. (2023). Precision Livestock Farming: What Does It Contain and What Are the Perspectives?. Animals.

[107] De Vries A., Bliznyuk N., Pinedo P. (2023). Invited Review: Examples and opportunities for artificial intelligence (AI) in dairy farms*. Appl. Anim. Sci..

[108] Fuentes S., Gonzalez Viejo C., Tongson E., Dunshea F.R. (2022). The livestock farming digital transformation: Implementation of new and emerging technologies using artificial intelligence. Anim. Health Res. Rev..

[109] Kaur U., Malacco V.M.R., Bai H., Price T.P., Datta A., Xin L., Sen S., Nawrocki R.A., Chiu G., Sundaram S. (2023). Invited review: Integration of technologies and systems for precision animal agriculture-a case study on precision dairy farming. J. Anim. Sci..

[110] Neethirajan S. (2023). Artificial Intelligence and Sensor Technologies in Dairy Livestock Export: Charting a Digital Transformation. Sensors.

[111] Aquilani C., Confessore A., Bozzi R., Sirtori F., Pugliese C. (2022). Review: Precision Livestock Farming technologies in pasture-based livestock systems. Animal.

[112] Karimuribo E.D., Batamuzi E.K., Massawe L.B., Silayo R.S., Mgongo F.O.K., Kimbita E., Wambura R.M. (2016). Potential use of mobile phones in improving animal health service delivery in underserved rural areas: Experience from Kilosa and Gairo districts in Tanzania. BMC Vet. Res..

[113] Bernabucci G., Evangelista C., Girotti P., Viola P., Spina R., Ronchi B., Bernabucci U., Basirisco L., Turini L., Mantino A. (2025). Precision livestock farming: An overview on the application in extensive systems. Ital. J. Anim. Sci..

[114] Rosati A. (2025). Guiding principles of AI: Application in animal husbandry and other considerations. Anim. Front..

